# Accelerometer-measured postoperative physical activity confers a significant mortality benefit following joint arthroplasty

**DOI:** 10.1302/2046-3758.156.BJR-2025-0684.R1

**Published:** 2026-06-18

**Authors:** Omar Musbahi, Sara Sousi, Ahmed Al-Saadawi, Sevasti Panagiota Glynou, Alex Bottle, Gareth Jones, Justin Cobb

**Affiliations:** 1 MSk Lab, Sir Michael Uren Hub, White City Campus, Imperial College London, London, UK; 2 School of Public Health, White City Campus, Imperial College London, London, UK; 3 Lister Hospital, East and North Hertfordshire Teaching NHS Trust, Stevenage, UK

**Keywords:** Accelerometer, Physical activity, Arthroplasty, Mortality, accelerometers, joint arthroplasty, cadence, knee arthroplasty procedures, hip and knee arthroplasty, Cox regression analysis, hip, retrospective cohort study, total knee arthroplasty (TKA), unicompartmental knee arthroplasty (UKA)

## Abstract

**Aims:**

The aim of this study was to examine the association between postoperative physical activity and mortality following hip and knee arthroplasty.

**Methods:**

A retrospective cohort study was conducted using the UK Biobank dataset, linked to hospital and death registry data. Adults undergoing hip or knee arthroplasty with valid postoperative accelerometer data were included. Physical activity (PA) metrics, including daily step count, light PA, moderate-to-vigorous PA, Euclidean Norm Minus One (ENMO), and peak cadence, were derived using a machine-learning algorithm. Survival probability was assessed up to 20 years postoperatively using Cox regression models.

**Results:**

A total of 1,769 patients were included. Between the ten- and 20-year follow-ups, higher PA was associated with reduced all-cause mortality. Multiple Cox regression highlighted that adjusted daily step count (HR 0.92, 95% CI 0.87 to 0.97; p = 0.004), light PA (HR 0.86, 95% CI 0.760 to 0.98; p = 0.020), moderate-to-vigorous PA (HR 0.64, 95% CI 0.42 to 0.97; p = 0.036), ENMO (HR 0.97, 95% CI 0.94 to 1.00; p = 0.030), and peak cadence (HR 0.98, 95% CI 0.97 to 0.99; p = 0.001) were significantly associated with reduced all-cause mortality.

**Conclusion:**

This is the first study to demonstrate that objectively measured postoperative PA predicts long-term survival following hip and knee arthroplasty. These findings highlight the importance of PA and careful procedure selection in maximizing long-term outcomes.

Cite this article: *Bone Joint Res* 2026;15(6):705–715.

## Article focus

- To investigate the association between objectively measured postoperative physical activity and long-term all-cause mortality following lower limb joint arthroplasty.

## Key messages

- Higher postoperative physical activity, as captured by wrist-worn accelerometers, was significantly associated with reduced long-term mortality following hip and knee arthroplasty.
- Patients undergoing hip resurfacing and unicompartmental knee arthroplasty demonstrated lower mortality than those undergoing total joint replacements.

## Strengths and limitations

- This is the first national-level study to link objective, accelerometer-measured post-operative physical activity with mortality over a 20-year follow-up period.
- Limitations include the absence of preoperative physical activity data and potential selection bias given the UK Biobank’s predominantly white cohort.

## Introduction

Globally, osteoarthritis affects over 7% of the population, and is a leading cause of hip and knee disability.^1^ Its management remains an area of active research interest, but initially involves a combination of pharmacological and non-pharmacological interventions aimed at optimizing symptom control.^2,3^ For patients with end-stage disease, joint arthroplasty is typically required, offering transformative outcomes, including restored mobility, pain relief, and ultimately enhanced quality of life.^2,4^ In the UK, an ageing population has driven increasing hip and knee arthroplasty rates, with forecasts predicting up to a 40% increase by 2060.^5^

Despite its well-established benefits, lower limb joint arthroplasty is not without complications, which may include infection, thromboembolism, periprosthetic fractures, cardiovascular events, and mortality.^6,7^ On the whole, mortality secondary to arthroplasty is a rare occurrence, but it remains a critical, routinely assessed postoperative outcome.^8^ A systematic review by Pan et al^9^ reported a 30-day mortality of 0.14% (95% CI 0.05 to 0.22) following total knee arthroplasty (TKA), which increased to 0.35% (95% CI 0.28 to 0.43) at 90 days and 1.1% (95% CI 0.71 to 1.49) at one year postoperatively. Mortality outcomes following total hip arthroplasty (THA) were comparable, with another systematic review by Turan et al^10^ reporting pooled rates of 0.49% (95% CI 0.23 to 0.84), 0.47% (95% CI 0.38 to 0.57), and 1.90% (95% CI 1.22 to 2.73) at 30 days, 90 days, and one year postoperatively, respectively. Factors associated with mortality following hip and knee arthroplasty include older age, male sex, BMI, and a range of comorbidities, such as cardiovascular and cerebrovascular disease.^11^

Another variable hypothesized to influence mortality outcomes is postoperative physical activity (PA) levels. Patients with end-stage osteoarthritis are often burdened with chronic comorbidities, with 85% experiencing at least one comorbidity and 29% experiencing more than three.^12^ Regular PA is known to play a protective role against the development or exacerbation of chronic health conditions; in this context, inactivity may accelerate the progression of such comorbidities, ultimately raising mortality risk.^13^

To date, no study has directly examined the association between PA levels and mortality outcomes following lower limb joint arthroplasty. Additionally, existing research on mortality following arthroplasty primarily focuses on short-term outcomes, with few studies reporting data up to a decade postoperatively. Hence, the aim of this study is to investigate the role of postoperative PA levels, measured via accelerometry, on mortality outcomes across TKA, unicompartmental knee arthroplasty (UKA), THA, and hip resurfacing arthroplasty (HRA) using data from the UK Biobank (UKB) registry.

## Methods

### Study design and data source

This study employed a retrospective cohort design using data from the UKB accelerometer dataset. The UKB is a large-scale database containing in-depth health information from half a million UK volunteers aged 40 to 69 years at recruitment (March 2006 to August 2010). Between 2013 and 2015, around 20% of the participants (n = 103,620) wore wrist-worn triaxial accelerometers (Axivity AX3; UK) for seven consecutive days.^14^ The accelerometry data was linked to Hospital Episode Statistics and death data, allowing for the identification of participants who underwent primary hip or knee arthroplasty procedures. The UKB study received ethical approval from the North West Multicentre Research Ethics Committee (REC reference: 16/NW/0274). This study was conducted and reported in accordance with the Strengthening the Reporting of Observational Studies in Epidemiology (STROBE) guidelines.

### Study population and eligibility criteria

Participants were included in the study if they had undergone primary unilateral TKA, UKA, THA, or HRA procedures, and had valid accelerometry data available at any postoperative timepoint. The identification of arthroplasty procedures was performed using relevant OPCS Classification of Interventions and Procedures version 4 (OPCS-4) codes from linked Hospital Episode Statistics.

Patients undergoing unilateral primary knee or hip arthroplasty were included; those undergoing bilateral joint arthroplasty or revision arthroplasty were excluded, as were any with missing key clinical and demographic variables. As such, this study is a complete case analysis. Only those with good calibration and wear time of the accelerometer were kept, as defined by the UKB database.^14,15^ The OxWearables step count package (v3.11.0; GitHub; Wearables Group, University of Oxford, UK)^16^ was used to extract the PA outcomes; it uses a self-supervised machine-learning algorithm tailored for wrist-worn accelerometer data, trained and fine-tuned on data from the Axivity AX3 device used by the UKB. The algorithm detects walking periods, estimates step counts, and calculates PA measures, expressed as the number of hours per day spent in each activity category alongside total step count. The variables used were the adjusted daily step count, peak adjusted cadence, light PA, moderate-to-vigorous PA (MVPA), and Euclidean Norm Minus One (ENMO), a measure of movement intensity. Specifically, peak cadence refers to the maximum average number of steps per minute achieved during the accelerometer period. Adjusted estimates account for missing accelerometer data using imputation based on time of day, whereby missing values are replaced with the mean from the corresponding timepoint on other valid days, subject to predefined wear-time thresholds. PA was assessed at a single postoperative timepoint using wrist-worn accelerometry, performed at a variable interval following surgery. Participants were not followed longitudinally with repeated accelerometer assessments. PA measures were therefore analyzed in relation to subsequent mortality status at predefined follow-up horizons (five, ten, 15, and 20 years), rather than representing activity levels measured at those postoperative timepoints.

### Statistical and survival analysis

Survival time was defined as the duration from the date of primary joint arthroplasty to either the recorded date of death or the end of the follow-up period, which was set as 31 December 2023. All-cause mortality was considered, and survival was assessed at five-, ten-, 15-, and 20-year intervals; the minimum survival time observed was greater than 365 days.

Bivariate analyses were conducted to explore differences in clinical, demographic, and PA characteristics across mortality intervals. Categorical variables were summarized as counts and percentages, while continuous variables were reported as medians with IQRs. Comparisons of continuous variables were made using the Mann-Whitney U test, due to non-normal distribution. For categorical data, the chi-squared test was used; Fisher’s exact test was applied where expected cell counts were less than five.

To further identify factors associated with long-term mortality, multiple logistic regression analyses were performed, adjusting for age at operation, sex, surgery type, time from surgery to accelerometer, Charlson Comorbidity Index (CCI) score,^17^ and smoking status.

Survival analysis was conducted using Cox proportional hazards regression to evaluate associations between clinical, demographic, and PA variables and mortality risk. Both unadjusted and adjusted models (same variables as for logistic regression) were constructed for each of the five PA outcomes: daily step count, MVPA, light PA, ENMO, and peak cadence. The proportional hazards assumption was tested using Schoenfeld residuals. This assumption was violated for the time interval between surgery and accelerometer wear; therefore, this covariate was modelled as a time-varying coefficient using a log-transformed function of time.

All analyses, including data preprocessing and visualizations, were performed in R (v. 4.4.1, R Foundation for Statistical Computing, Austria). Statistical significance was set at *α* = 0.05, unless otherwise specified.

## Results

**Fig. 1:**
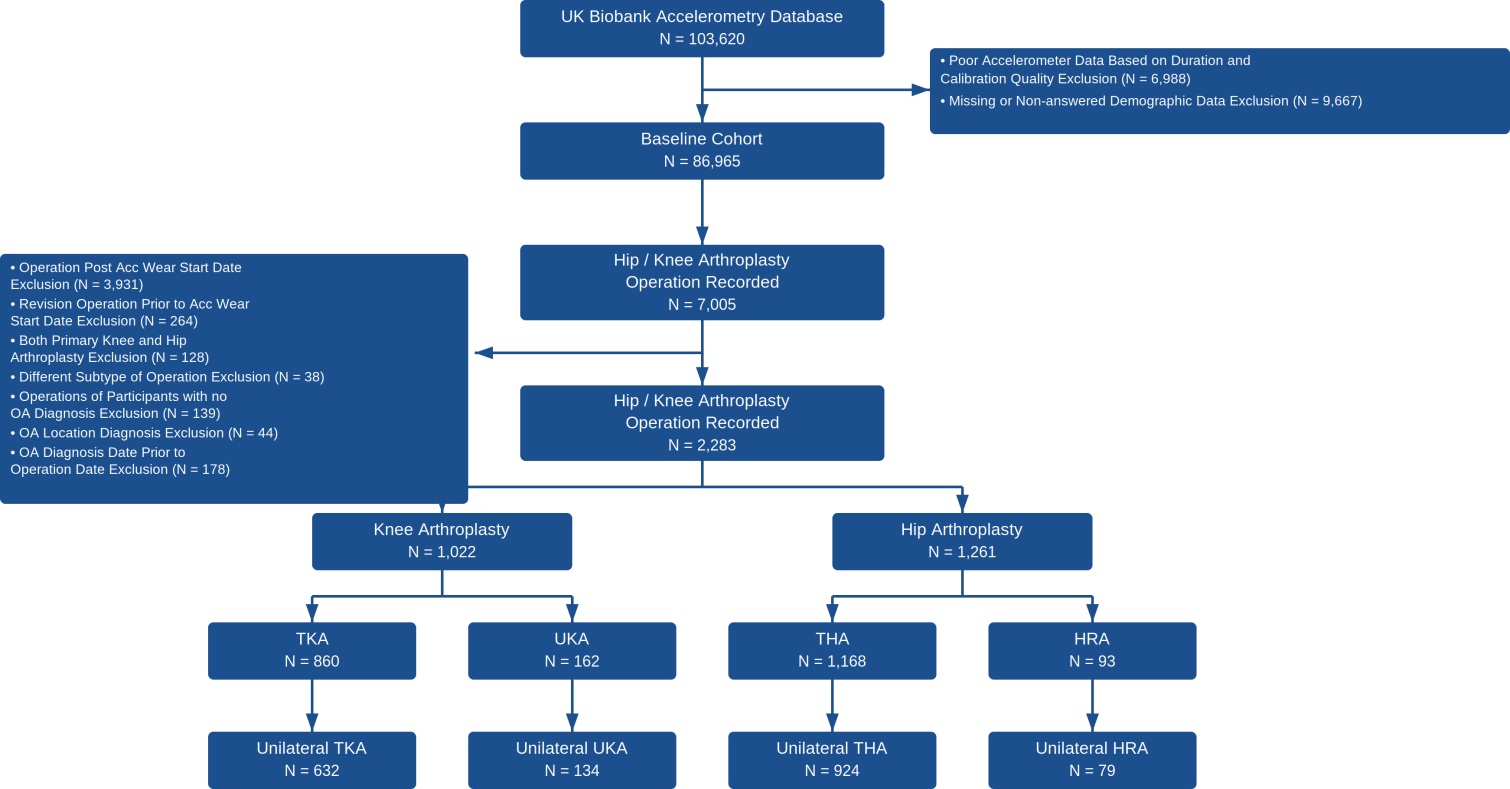
Patient inclusion criteria workflow. HRA, hip resurfacing arthroplasty; OA, osteoarthritis; THA, total hip arthroplasty; TKA, total knee arthroplasty; UKA, unicompartmental knee arthroplasty.

A total of 1,769 patients met the inclusion criteria and were included in the final analysis. Of these, 632 patients underwent TKA, 134 UKA, 924 THA, and 79 HRA (Figure 1).

Patient demographic data and PA metrics were compared between alive and deceased patients. A significant difference in the mean time from surgery to accelerometer data was noted at five-year (alive vs deceased: 3.3 vs 1.3; p < 0.001, Mann-Whitney U test) and ten-year (3.3 vs 2.0; p < 0.001, Mann-Whitney U test) follow-up intervals. At the five-year follow-up, 19 patients from the original cohort had died, with no significant difference in mortality observed between the individual procedures (p = 0.154, chi-squared test). However, a statistically significant difference was elicited at the later follow-up intervals (ten years: p = 0.016, chi-squared test). The median age of alive patients at the five-year follow-up interval was 64 years (IQR 59 to 68), which was significantly lower than the median age of deceased patients, at 68 years (IQR 61 to 71). This significant difference persisted at ten-year (p < 0.001), 15-year (p < 0.001), and 20-year (p = 0.002) follow-up intervals. Furthermore, a significant difference in sex distribution was also observed across all follow-up intervals, with males accounting for 79% (15/19) of deaths at the five-year follow-up (p < 0.001, chi-squared test). Additionally, there were no differences in BMI (p = 0.623, Mann-Whitney U test) or ethnicity (p > 0.999, Mann-Whitney U test) at the initial five-year follow-up. The lack of significant difference in ethnicity between alive and deceased patients persisted through to the final 20-year postoperative follow-up. However, BMI rates became significantly different between the two groups at the 15-year (p = 0.002, Mann-Whitney U test) and 20-year (p = 0.002, Mann-Whitney U test) follow-up timepoints. CCI score was significantly different across all timepoints (five years: p = 0.008, chi-squared test). A significant difference in smoking habits was also observed between the two groups across all follow-up intervals (five years: p = 0.018, chi-squared test), whereas differences in alcohol consumption only reached statistical significance at the 15-year (p = 0.016, chi-squared test) and 20-year (p = 0.019, chi-squared test) follow-up timepoints. The remaining patient characteristics can be seen in Table I.

**Table I.** Clinical and demographic data of patients undergoing primary lower limb joint arthroplasty stratified by mortality status.

Variable	5-year mortality			10-year mortality			15-year mortality			20-year mortality		
	Alive n = 1,750	Dead n = 19	p-value	Alive n = 1,700	Dead n = 69	p-value	Alive n = 1,670	Dead n = 99	p-value	Alive n = 1,658	Dead n = 111	p-value
**Surgery type, n (%)**			0.154*			0.016*			0.001*			0.003*
HRA	79 (4.5)	0 (0)		79 (4.6)	0 (0)		78 (4.7)	1 (1.0)		78 (4.7)	1 (0.9)	
THA	918 (52)	6 (32)		895 (53)	29 (42)		887 (53)	37 (37)		879 (53)	45 (41)	
TKA	621 (35)	11 (58)		596 (35)	36 (52)		580 (35)	52 (53)		576 (35)	56 (50)	
UKA	132 (7.5)	2 (11)		130 (7.6)	4 (5.8)		125 (7.5)	9 (9.1)		125 (7.5)	9 (8.1)	
**Median age at surgery, yrs (IQR)**	64 (59 to 68)	68 (61 to 71)	0.034†	64 (59 to 68)	68 (64 to 71)	< 0.001†	64 (59 to 68)	66 (63 to 70)	< 0.001†	64 (59 to 68)	65 (62 to 69)	0.002†
**Sex, n (%)**			< 0.001*			0.001*			< 0.001*			< 0.001*
Female	1,024 (59)	4 (21)		1,001 (59)	27 (39)		993 (59)	35 (35)		988 (60)	40 (36)	
Male	726 (41)	15 (79)		699 (41)	42 (61)		677 (41)	64 (65)		670 (40)	71 (64)	
**Median BMI, kg/m² (IQR)**	27.8 (25.0 to 31.3)	29.4 (25.5 to 31.1)	0.623†	27.7 (25.0 to 31.2)	29.9 (25.6 to 32.4)	0.059†	27.7 (24.9 to 31.2)	30.0 (25.9 to 33.1)	0.002†	27.7 (24.9 to 31.2)	29.9 (25.9 to 32.8)	0.002†
**Ethnicity, n (%)**			> 0.999‡			0.622‡			0.395‡			0.400‡
Non-white	25 (1.4)	0 (0)		25 (1.5)	0 (0)		25 (1.5)	0 (0)		25 (1.5)	0 (0)	
White	1,725 (99)	19 (100)		1,675 (99)	69 (100)		1,645 (99)	99 (100)		1,633 (98)	111 (100)	
**CCI score, n (%)**			0.008*			< 0.001*			< 0.001*			< 0.001*
0	1,412 (81)	12 (63)		1,379 (81)	45 (65)		1,356 (81)	68 (69)		1,346 (81)	78 (70)	
1	220 (13)	2 (11)		210 (12)	12 (17)		206 (12)	16 (16)		206 (12)	16 (14)	
2	79 (4.5)	2 (11)		76 (4.5)	5 (7.2)		75 (4.5)	6 (6.1)		74 (4.5)	7 (6.3)	
3+	39 (2.2)	3 (16)		35 (2.1)	7 (10)		33 (2.0)	9 (9.1)		32 (1.9)	10 (9.0)	
**Alcohol, n (%)**			0.262*			0.093*			0.016*			0.019*
Never	96 (5.5)	1 (5.3)		90 (5.3)	7 (10)		88 (5.3)	9 (9.1)		87 (5.2)	10 (9.0)	
< 3 d/w	759 (43)	5 (26)		741 (44)	23 (33)		734 (44)	30 (30)		729 (44)	35 (32)	
≥ 3 d/w	895 (51)	13 (68)		869 (51)	39 (57)		848 (51)	60 (61)		842 (51)	66 (59)	
**Smoking, n (%)**			0.018*			0.012*			< 0.001*			< 0.001*
Never	921 (53)	5 (26)		899 (53)	27 (39)		892 (53)	34 (34)		885 (53)	41 (37)	
Previous	742 (42)	11 (58)		719 (42)	34 (49)		698 (42)	55 (56)		695 (42)	58 (52)	
Current	87 (5.0)	3 (16)		82 (4.8)	8 (12)		80 (4.8)	10 (10)		78 (4.7)	12 (11)	
**Education, n (%)**			0.691*			0.861*			0.836*			0.571*
Further education	730 (42)	10 (53)		712 (42)	28 (41)		699 (42)	41 (41)		692 (42)	48 (43)	
Higher education	685 (39)	6 (32)		662 (39)	29 (42)		650 (39)	41 (41)		645 (39)	46 (41)	
School leaver	335 (19)	3 (16)		326 (19)	12 (17)		321 (19)	17 (17)		321 (19)	17 (15)	
**Townsend Deprivation index, n (%)**			0.279*			0.583*			0.217*			0.279*
1	448 (26)	5 (26)		437 (26)	16 (23)		434 (26)	19 (19)		428 (26)	25 (23)	
2	406 (23)	2 (11)		390 (23)	18 (26)		380 (23)	28 (28)		378 (23)	30 (27)	
3	350 (20)	2 (11)		342 (20)	10 (14)		337 (20)	15 (15)		337 (20)	15 (14)	
4	327 (19)	6 (32)		316 (19)	17 (25)		309 (19)	24 (24)		307 (19)	26 (23)	
5	219 (13)	4 (21)		215 (13)	8 (12)		210 (13)	13 (13)		208 (13)	15 (14)	
**Median time from surgery to accelerometer, yrs (IQR)**	3.3 (1.3 to 6.1)	1.3 (0.6 to 3.0)	< 0.001†	3.3 (1.3 to 6.3)	2.0 (0.9 to 3.6)	< 0.001†	3.3 (1.3 to 6.1)	3.1 (1.1 to 5.7)	0.489†	3.2 (1.3 to 6.0)	3.6 (1.4 to 7.2)	0.284†

* Pearson’s chi-squared test.

† Mann-Whitney U test.

‡ Fisher’s exact test, applied due to expected cell counts < 5.

CCI, Charlson Comorbidity Index; d/w, days/week; HRA, hip resurfacing arthroplasty; THA, total hip arthroplasty; TKA, total knee arthroplasty; UKA, unicompartmental knee arthroplasty.

Table II presents mortality rates at each interval, stratified by procedure type. The largest proportion of deaths occurred in patients undergoing TKA and UKA, with 1.7% and 1.5%, respectively, at five years, and increased to 8.9% and 6.7% at 20-year follow-up. THA mortality reached 4.9% at 20 years, while only a single death occurred in the HRA cohort (1.3%).

**Table II.** Mortality rates stratified by procedure type.

Mortality and procedure type	Total, n	Alive, n (%)	Dead, n (%)
**5-year mortality**			
HRA	79	79 (100)	0 (0)
THA	924	918 (99.4)	6 (0.6)
TKA	632	621 (98.3)	11 (1.7)
UKA	134	132 (98.5)	2 (1.5)
**10-year mortality**			
HRA	79	79 (100)	0 (0)
THA	924	895 (96.9)	29 (3.1)
TKA	632	596 (94.3)	36 (5.7)
UKA	134	130 (97.0)	4 (3.0)
**15-year mortality**			
HRA	79	78 (98.7)	1 (1.3)
THA	924	887 (96.0)	37 (4.0)
TKA	632	580 (91.8)	52 (8.2)
UKA	134	125 (93.3)	9 (6.7)
**20-year mortality**			
HRA	79	78 (98.7)	1 (1.3)
THA	924	879 (95.1)	45 (4.9)
TKA	632	576 (91.1)	56 (8.9)
UKA	134	125 (93.3)	9 (6.7)

HRA, hip resurfacing arthroplasty; THA, total hip arthroplasty; TKA, total knee arthroplasty; UKA, unicompartmental knee arthroplasty.

As seen in Table III, at the five-year follow-up, a significant difference in peak cadence was detected, favouring the alive group (p = 0.014), while the remaining PA metrics did not demonstrate any statistically significant difference. However, at the remaining follow-up intervals (ten, 15, and 20 years postoperatively), adjusted daily step count (ten years: p = 0.002; 15 years: p < 0.001; 20 years: p < 0.001), light PA (ten years: p = 0.005; 15 years: p < 0.001; 20 years: p < 0.001), MVPA (ten years: p = 0.004; 15 years: p = 0.002; 20 years: p = 0.005), ENMO (ten years: p = 0.004; 15 years: p < 0.001; 20 years: p < 0.001), and peak cadence (ten years: p < 0.001; 15 years: p < 0.001; 20 years: p < 0.001) were all significantly greater in the alive group compared with their counterparts.

**Table III.** Postoperative physical activity (PA) outcomes measured using wrist-worn accelerometers, stratified by subsequent mortality status at five-, ten-, 15-, and 20-year follow-up intervals. Physical activity was assessed at a single postoperative timepoint and does not represent longitudinal or contemporaneous measurements at each follow-up interval. Values are presented as medians (IQRs).

Mortality	Physical activity	Alive	Dead	Mean difference	95% CI	p-value*
5-year mortality	Adjusted daily step count	8,684 (6,328 to 11,123)	6,640 (3,382 to 10,136)	+ 1,485	−920 to 3,891	0.058
	Light PA, hrs/day	4.89 (3.88 to 6.01)	4.15 (2.86 to 6.17)	+ 0.68	−0.28 to 1.60	0.244
	MVPA, hrs/day	0.42 (0.17 to 0.80)	0.23 (0.01 to 0.66)	+ 0.20	−0.01 to 0.41	0.064
	ENMO	25 (21 to 30)	25 (15 to 32)	+ 1.8	−3.3 to 6.8	0.358
	Peak adjusted cadence	110 (102 to 118)	102 (93 to 112)	+ 9.6	0.55 to 19	0.014
10-year mortality	Adjusted daily step count	8,727 (6,401 to 11,128)	6,813 (4,673 to 9,793)	+ 1,427	387 to 2,467	0.002
	Light PA, hrs/day	4.90 (3.91 to 6.01)	4.25 (2.86 to 5.60)	+ 0.62	0.15 to 1.10	0.005
	MVPA, hrs/day	0.42 (0.18 to 0.81)	0.31 (0.07 to 0.65)	+ 0.17	0.06 to 0.28	0.004
	ENMO	25 (21 to 30)	23 (17 to 28)	+ 2.6	0.48 to 4.8	0.004
	Peak adjusted cadence	110 (102 to 118)	106 (97 to 113)	+ 6.6	2.7 to 10	< 0.001
15-year mortality	Adjusted daily step count	8,739 (6,426 to 11,140)	7,015 (4,673 to 9,649)	+ 1,516	693 to 2,338	< 0.001
	Light PA, hrs/day	4.91 (3.93 to 6.03)	4.24 (2.86 to 5.48)	+ 0.70	0.33 to 1.10	< 0.001
	MVPA, hrs/day	0.43 (0.18 to 0.81)	0.29 (0.08 to 0.65)	+ 0.14	0.04 to 0.25	0.002
	ENMO	25 (21 to 30)	22 (17 to 27)	+ 3.1	1.4 to 4.8	< 0.001
	Peak adjusted cadence	110 (102 to 118)	105 (96 to 113)	+ 6.6	3.3 to 9.8	< 0.001
20-year mortality	Adjusted daily step count	8,739 (6,426 to 11,140)	7,448 (4,681 to 9,793)	+ 1,326	547 to 2,106	< 0.001
	Light PA, hrs/day	4.91 (3.93 to 6.04)	4.30 (2.96 to 5.51)	+ 0.63	0.28 to 0.97	< 0.001
	MVPA, hrs/day	0.43 (0.18 to 0.81)	0.34 (0.08 to 0.66)	+ 0.11	0.01 to 0.22	0.005
	ENMO	25 (21 to 30)	22 (18 to 28)	+ 2.8	1.2 to 4.4	< 0.001
	Peak adjusted cadence	110 (102 to 118)	105 (96 to 113)	+ 6.2	3.1 to 9.2	< 0.001

* Mann-Whitney U test.

ENMO, Euclidean Norm Minus One; MVPA, moderate-to-vigorous physical activity.

Unadjusted logistic regression revealed age at surgery (odds ratio (OR) 1.09, p = 0.049), male sex (OR 5.29, p = 0.003), CCI score (OR 1.50, p = 0.002), being a current smoker (compared with never-smokers) (OR 6.35, p = 0.012), time from surgery to accelerometer (OR 0.66, p = 0.004), and adjusted peak cadence (OR 0.96, p = 0.001) as independent predictors of five-year mortality.

For ten-year mortality, independent predictors included THA (OR 0.54, p = 0.015), age at surgery (OR 1.11, p < 0.001), male sex (OR 2.23, p = 0.001), CCI score (OR 1.46, p < 0.001), current smoker (compared with never-smokers) (OR 3.25, p = 0.005), alcohol consumption less than three days per week (compared with never-drinkers) (OR 0.40, p = 0.039), time from surgery to accelerometer (OR 0.80, p < 0.001), light PA (OR 0.77, p = 0.002), MVPA (OR 0.49, p = 0.015), ENMO (OR 0.95, p = 0.005), daily adjusted step counts (OR 0.89, p = 0.002), and adjusted peak cadence (OR 0.97, p < 0.001).

Furthermore, THA (OR 0.47, p < 0.001), age at surgery (OR 1.08, p < 0.001), male sex (OR 2.68, p < 0.001), BMI (OR 1.05, p = 0.005), CCI score (OR 1.45, p < 0.001), current (OR 3.28, p = 0.002) and former smoker (OR 2.07, p = 0.002) (compared with never-smokers), alcohol consumption less than three days per week (OR 0.40, p = 0.021) (compared with never-drinkers), light PA (OR 0.75, p < 0.001), MVPA (OR 0.57, p = 0.016), daily adjusted step counts (OR 0.88, p < 0.001), ENMO (OR 0.94, p < 0.001), and adjusted peak cadence (OR 0.97, p < 0.001) were found to be independent predictors for 15-year mortality.

Lastly, THA (OR 0.53, p = 0.002), HRA (OR 0.13, p = 0.046), age at surgery (OR 1.06, p < 0.001), male sex (OR 2.62, p < 0.001), BMI (OR 1.05, p = 0.006), CCI score (OR 1.43, p < 0.001), current (OR 3.32, p < 0.001) and former smoker (OR 1.80, p = 0.005) (compared with never-smokers), alcohol consumption less than three days per week (OR 0.42, p = 0.021) (compared with never-drinkers), light PA (OR 0.77, p < 0.001), MVPA (OR 0.66, p = 0.046), daily adjusted step counts (OR 0.90, p < 0.001), ENMO (OR 0.94, p < 0.001), and adjusted peak cadence (OR 0.97, p < 0.001) were all independent predictors of 20-year mortality (Appendix A).

**Fig. 2:**
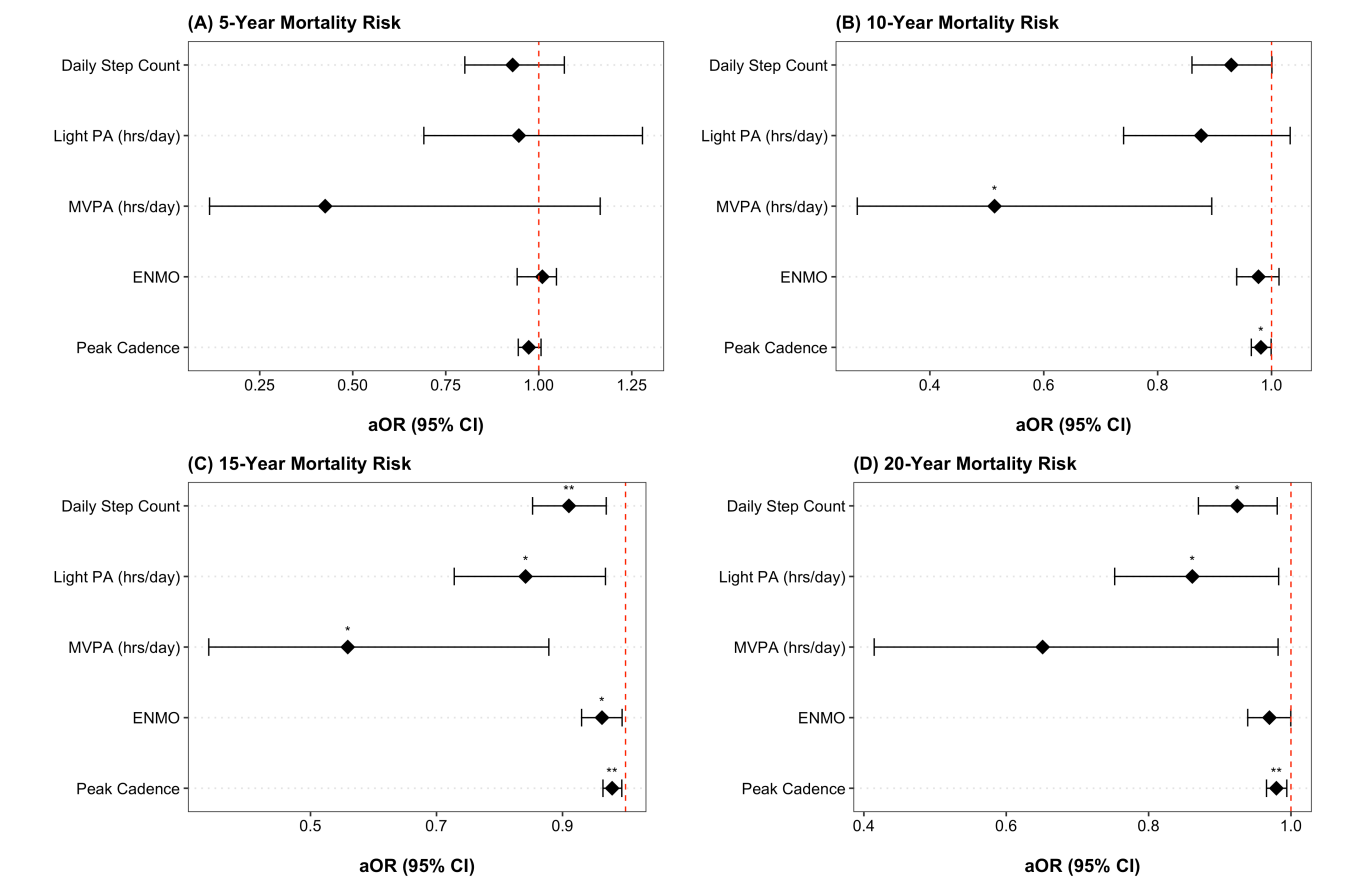
Association between physical activity (PA) measures and all-cause mortality risk at a) five years, b) ten years, c) 15 years, and d) 20 years. Forest plots display adjusted odds ratios (aORs) and 95% CIs for each PA metric, including daily step count, light PA, moderate-to-vigorous PA (MVPA), Euclidean Norm Minus One (ENMO), and peak cadence. Logistic regression models were adjusted for age at surgery, sex, procedure type (total hip arthroplasty, hip resurfacing, total knee arthroplasty, unicompartmental knee arthroplasty), time from surgery to accelerometer use, Charlson Comorbidity Index, and smoking status. The red dashed line indicates the null value (aOR = 1). Significance levels: *p < 0.05; **p < 0.01.

Figure 2 presents forest plots illustrating adjusted logistic regression ORs for each PA metric in relation to all-cause mortality. At the five-year follow-up, none of the PA metrics were associated with a significantly reduced all-cause mortality (Figure 2a). Peak cadence gained a statistically significant association with all-cause mortality at the ten-year follow-up (p < 0.036), while the remaining metrics remained non-significant (Figure 2b). Furthermore, daily step count (p < 0.004), light PA (p < 0.017), MVPA (p < 0.017), ENMO (p < 0.026), and peak cadence (p < 0.006) all demonstrated a significant association with 15-year all-cause mortality (Figure 2c). Daily step count (p < 0.010), light PA (p < 0.028), and peak cadence (p < 0.005) remained significantly associated with all-cause mortality at the 20-year follow-up, whereas ENMO no longer reached statistical significance (Figure 2d). The complete set of ORs from the multiple logistic regression analysis of PA and various patient demographics associated with all-cause mortality can be seen in Appendices B to F.

Unadjusted Cox regression analysis revealed that adjusted daily step count (hazard ratio (HR) 0.90, 95% CI 0.85 to 0.95; p < 0.001), light PA (HR 0.78, 95% CI 0.69 to 0.88; p < 0.001), MVPA (HR 0.75, 95% CI 0.44 to 0.98; p = 0.039), ENMO (HR 0.94, 95% CI 0.92 to 0.97; p < 0.001), and adjusted peak cadence (HR 0.97, 95% CI 0.96 to 0.98; p < 0.001) were significantly associated with improved overall survival. In terms of clinical and demographic variables, age at surgery (HR 1.11, 95% CI 1.07 to 1.15; p < 0.001), male sex (HR 2.47, 95% CI 1.68 to 3.62; p < 0.001), BMI (HR 1.05, 95% CI 1.02 to 1.09; p = 0.004), CCI score (HR 1.38, 95% CI 1.23 to 1.55; p < 0.001), and current (HR 3.24, 95% CI 1.71 to 6.16; p < 0.001) and former smokers (HR 1.79, 95% CI 1.20 to 2.66; p = 0.004) (compared with never-smokers) were associated with an increased all-cause mortality risk. In contrast, THA (HR 0.50, 95% CI 0.34 to 0.75; p < 0.001), HipR (HR 0.09, 95% CI 0.01 to 0.63; p < 0.016), and alcohol consumption less than three days per week (HR 0.47, 95% CI 0.23 to 0.96; p = 0.037) decreased the risk of all-cause mortality following arthroplasty (Appendix G).

Consistent with the unadjusted analysis, multiple Cox regression demonstrated that adjusted daily step count (HR 0.92, 95% CI 0.87 to 0.97; p = 0.004), light PA (HR 0.86, 95% CI 0.76 to 0.98; p = 0.020), MVPA (HR 0.64, 95% CI 0.42 to 0.97; p = 0.036), ENMO (HR 0.97, 95% CI 0.94 to 1.00; p = 0.030), and peak cadence (HR 0.98, 95% CI 0.97 to 0.99; p = 0.001) were all significantly associated with reduced all-cause mortality following lower limb joint arthroplasty. The HRs and corresponding p-values for the remaining clinical and demographic variables can be seen in Table IV.

**Table IV.** Multiple Cox regression analysis for overall survival stratified by each physical activity (PA) measure derived by accelerometer data.

Variable	Daily step count (per 1,000)		MVPA (hrs/day)		Light PA (hrs/day)		ENMO acceleration		Peak cadence (steps/min)	
	HR (95% CI)	p-value	HR (95% CI)	p-value	HR (95% CI)	p-value	HR (95% CI)	p-value	HR (95% CI)	p-value
PA measure	0.92 (0.87 to 0.97)	0.004	0.64 (0.42 to 0.97)	0.036	0.86 (0.76 to 0.98)	0.020	0.97 (0.94 to 1.00)	0.030	0.98 (0.97 to 0.99)	0.001
Age at surgery, yrs	1.07 (1.03 to 1.12)	< 0.001	1.08 (1.03 to 1.12)	< 0.001	1.08 (1.03 to 1.12)	< 0.001	1.07 (1.03 to 1.12)	0.001	1.08 (1.03 to 1.12)	< 0.001
**Sex**										
Female	—		—		—		—		—	
Male	2.51 (1.69 to 3.72)	< 0.001	2.62 (1.75 to 3.93)	< 0.001	2.11 (1.42 to 3.14)	< 0.001	2.33 (1.58 to 3.45)	< 0.001	2.30 (1.56 to 3.40)	< 0.001
**Surgery type**										
TKA	—		—		—		—		—	
UKA	0.93 (0.46 to 1.90)	0.844	0.86 (0.42 to 1.74)	0.671	0.85 (0.42 to 1.73)	0.653	0.84 (0.41 to 1.71)	0.636	0.91 (0.45 to 1.85)	0.792
THA	0.18 (0.02 to 1.31)	0.090	0.17 (0.02 to 1.24)	0.080	0.15 (0.02 to 1.07)	0.059	0.16 (0.02 to 1.17)	0.072	0.18 (0.02 to 1.29)	0.087
HRA	0.70 (0.47 to 1.04)	0.079	0.68 (0.46 to 1.02)	0.060	0.67 (0.45 to 1.00)	0.050	0.68 (0.46 to 1.02)	0.063	0.71 (0.48 to 1.06)	0.093
Time from surgery to accelerometer, yrs*	0.99 (0.98 to 1.00)	0.149	0.99 (0.98 to 1.00)	0.167	0.99 (0.98 to 1.00)	0.191	0.99 (0.98 to 1.00)	0.150	0.99 (0.98 to 1.00)	0.182
**CCI score**	1.35 (1.18 to 1.54)	< 0.001	1.35 (1.19 to 1.54)	< 0.001	1.37 (1.20 to 1.55)	< 0.001	1.35 (1.19 to 1.54)	< 0.001	1.36 (1.19 to 1.54)	< 0.001
**Smoking**										
Never	—		—		—		—		—	
Previous	1.40 (0.93 to 2.09)	0.103	1.40 (0.93 to 2.09)	0.103	1.44 (0.97 to 2.16)	0.073	1.43 (0.96 to 2.14)	0.082	1.36 (0.91 to 2.03)	0.135
Current	3.29 (1.72 to 6.29)	< 0.001	3.40 (1.78 to 6.50)	< 0.001	3.45 (1.80 to 6.58)	< 0.001	3.38 (1.77 to 6.47)	< 0.001	3.06 (1.59 to 5.87)	< 0.001

* Time from surgery to accelerometer worn is modelled as a time-varying coefficient using a log-transformed function of time.

CCI, Charlson Comorbidity Index; ENMO, Euclidean Norm Minus One; MVPA, moderate-to-vigorous physical activity; THA, total hip arthroplasty; TKA, total knee arthroplasty; UKA, unicompartmental knee arthroplasty.

**Fig. 3:**
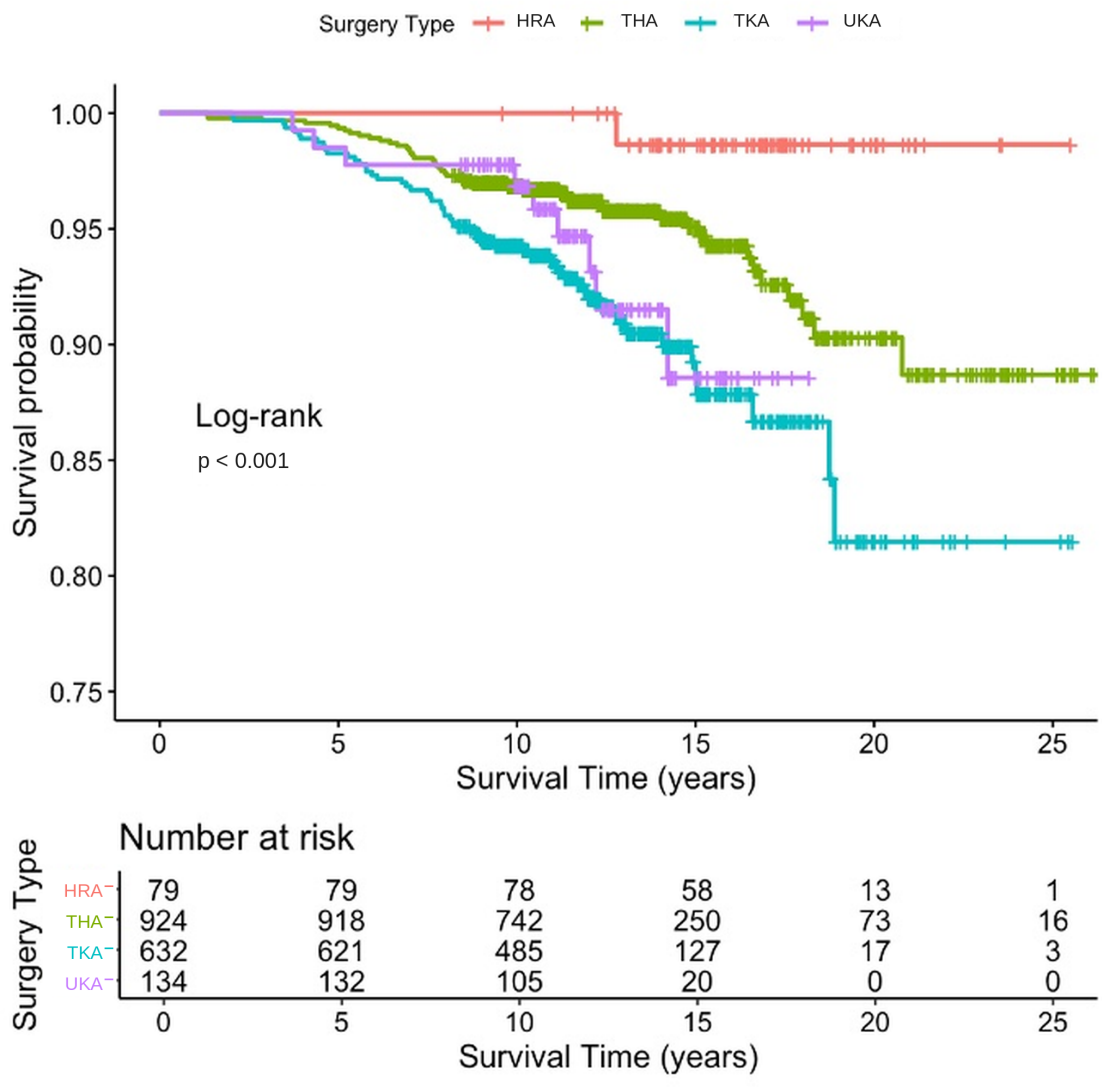
Kaplan-Meier analysis for overall survival of patients undergoing primary joint arthroplasty stratified by surgery type. HRA, hip resurfacing; THA, total hip arthroplasty; TKA, total knee arthroplasty; UKA, unicompartmental knee arthroplasty.

As seen in Figure 3, Kaplan-Meier survival analysis demonstrated statistically significant differences in survival probabilities between THA, HipR, TKA, and UKA (p < 0.001). HipR exhibited the greatest survival probability among the procedures, followed by THA. Conversely, TKA was associated with the lowest survival probabilities.

## Discussion

To the best of our knowledge, no study has directly examined the predictive power of postoperative PA and all-cause mortality following lower limb joint arthroplasty. Our study confirms that all objectively measured parameters of PA (light PA, MVPA, ENMO, peak cadence) measured following recovery from surgery were significantly associated with reduced mortality starting from ten years postoperatively, sustained through to the final follow-up at 20 years. Additionally, when each procedure was analyzed individually, both THA and HRA incurred significantly lower mortality rates than TKA, as demonstrated on unadjusted Cox regression and Kaplan-Meier survival analysis. Lastly – in addition to PA – age, male sex, BMI, CCI score, and alcohol and smoking habits were all found to be significant independent predictors of postoperative mortality.

Our findings align with the current literature regarding the impact of PA on all-cause mortality outcomes following lower limb joint arthroplasty. Klimek et al^18^ reported that functional impairment, using the Hanover Functionality Status Questionnaire, was found to be an independent predictor of one-year mortality (HR 1.79, 95% CI 1.24 to 2.60; p < 0.01) following TKA and THA. However, this metric is self-reported and, while related, does not directly assess PA. Additionally, their study cohort was limited to patients from four institutions in southwest Germany.^18^ Conversely, our study uses a wider, more diverse dataset spanning across the entire UK, thereby enhancing the generalizability of our findings. Another study, by Jämsen et al,^19^ found that preoperative mobility restrictions were significantly associated with a higher five-year mortality risk following TKA and THA, in agreement with our postoperative findings. Again, PA was not directly measured, but instead a clinician-led mobility rating scale was used, which introduces a degree of bias. It also focuses on preoperative functional status, rather than our focus of objectively measured postoperative PA.^19^ While pre- and postoperative functional status are related, their clinical significance differs considerably: preoperative status is often viewed as a reflection of surgical risk, while postoperative status serves as a predictor of recovery outcome. In this context, while our results are in agreement, the validity of such comparisons is limited.

Several aspects of our study distinguish it from prior research. First, it uses long-term mortality follow-up data, extending up to 20 years, offering a comprehensive overview of mortality following arthroplasty and the key factors influencing it. Second, our inclusion of UKA and HRA provides unique insights into post-arthroplasty outcomes. Third, this study used numerous objectively measured metrics from a wrist-worn accelerometer as outcome measures, in contrast to many previous studies which relied solely on either patient-reported outcome measures or simple activity minutes to assess functional performance.^20-22^ While we acknowledge that patient-reported outcome measures are an important method of assessment, a degree of bias exists within their application, and our outcome measures overcome these limitations, ultimately providing more accurate results.^23^

Despite the novelty of our findings, several limitations should be acknowledged. Specifically, no data regarding patient selection were available, so any conclusions must be cautious, despite earlier work from our group suggesting that procedure selection is dominated by surgeon choice rather than disease severity. Moreover, our cohort consisted of predominantly white and well-educated patients. This lack of diversity limits the generalizability and subsequent external validity of our findings. As accelerometer data were collected at a single postoperative timepoint, age-related or time-dependent declines in PA could not be assessed, and the observed associations reflect the prognostic value of early postoperative activity rather than sustained activity across the lifespan. Lastly, without preoperative accelerometer data,^24^ any conclusions are limited, but the size of the protective effect of the two less radical procedures on mortality is exactly at odds with the reported ‘success’ of total joint replacement when measured by revision rate alone.

This study is the first to examine the association between postoperative PA and all-cause mortality following lower limb joint arthroplasty. All PA parameters postoperatively were associated with a significantly reduced mortality, beginning at ten years postoperatively and sustained through to the final 20-year follow-up. As expected, clinical and demographic factors, such as age, male sex, procedure type, BMI, smoking status, and alcohol consumption, were independently associated with all-cause mortality following arthroplasty. The less radical procedures of partial knee arthroplasty and hip resurfacing appear to offer some reduction in mortality, which may be related to the levels of activity reported following these less invasive procedures; however, selection bias remains a possible contributor. Future research should focus on identifying the pre- and perioperative factors which may have a causative impact on the level of postoperative PA sufficient to confer improved survival outcomes following any form of arthroplasty. Alongside this, longitudinal accelerometer data should also be incorporated into future research to better characterize age-related trajectories in PA and their subsequent influence on long-term mortality outcomes.

## Data Availability

The datasets generated and analyzed in the current study are not publicly available due to data protection regulations. Access to the data is made to any researcher via the UK Biobank policy. Access to data is limited to the researchers who have obtained permission for data processing. Further inquiries can be made to the corresponding author.
